# Effect of Modified Roux-en-Y Gastric Bypass Surgery on GLP-1, GIP in Patients with Type 2 Diabetes Mellitus

**DOI:** 10.1155/2015/625196

**Published:** 2015-06-18

**Authors:** Shao-Wei Xiong, Jing Cao, Xian-Ming Liu, Xing-Ming Deng, Zeng Liu, Fang-Ting Zhang

**Affiliations:** ^1^Department of General Surgery, Peking University Shenzhen Hospital, Shenzhen, Guangdong 518036, China; ^2^Central Laboratory, Peking University Shenzhen Hospital, Shenzhen, Guangdong 518036, China

## Abstract

The type 2 diabetes mellitus (T2DM) is one of the most serious diseases that threaten public health. Modified gastric bypass surgery has been applied to the treatment of T2DM patients in the 1990s, but the therapeutic mechanism to this function is still unclear. The aim of this study was to further clarify the effect and the mechanism of modified gastric bypass surgery on glucose metabolism in patients with T2DM. In the study, the incretin indexes and blood glucose indexes were analyzed before surgery and 1 week and 1, 3, and 6 months after surgery. The results suggested that modified Roux-en-Y gastric bypass can promote GLP-1 secretion in patients with T2DM, while reducing the secretion of GIP. Thus it could effectively control blood glucose of patients with T2DM.

## 1. Introduction

The type 2 diabetes mellitus (T2DM) is a kind of commonly and frequently encountered disease which severely threatens people's health. Completely cured T2DM is a difficult problem on medicine. Modified gastric bypass surgery began to be used in T2DM patients in the 1990s [1, 2], and the efficiency of this procedure reached 83% [3–10] although the therapeutic mechanism involved is still unclear. In order to further clarify the effect and the mechanism of modified gastric bypass surgery on glucose metabolism in patients with T2DM, we retrospectively analyzed incretin indexes including GLP-1, GIP, and some blood glucose indexes in patients with T2DM underwent modified Roux-en-Y gastric bypass procedures.

## 2. Materials and Methods

### 2.1. General Data

A retrospective analysis of the clinical data of 50 patients with T2DM and gastric cancer or simple T2DM treated in the department of general surgery from January 2006 to September 2014 was conducted. The patients underwent gastrectomy using modified Roux-en-Y gastric bypass procedures. Follow-up data were complete. The patients in this study were diagnosed with T2DM in accordance with the diagnostic criteria of the American Diabetes Association (2009). Specific parameters measured were as follows: symptoms of diabetes mellitus, random blood glucose ≥11.1 mmol/L, fasting plasma glucose ≥7.0 mmol/L, and an oral glucose tolerance test at 2 h ≥11.1 mmol/L. If the patients met one of the above conditions and retest the following day conformed to the standards of the diagnosis of diabetes, the patient was then diagnosed with diabetes mellitus. Islet cell antibodies, antibody to insulin, glutamic acid decarboxylase antibody, glycosylated hemoglobin (HbA1c), and C peptide level were examined to define T2DM.

In 50 patients, 27 patients were males, the rest 23 are female, the average age is 46.18 + 11.36 years (the age range is 30–66 years), and the mean BMI is 25.23 + 1.21 kg/square meters.

### 2.2. Surgery

Patients with T2DM and gastric cancer were treated with radical resection of gastric cancer firstly. According to the location of cancer in the gastric cavity, the patients underwent radical total gastrectomy or distal gastrectomy (residual gastric cavity volume 30–50 ML), stomach perigastric lymph node dissection. Simple T2DM patients were treated with cutting transversely in the upper gastric body to close the gastric cavity, residual gastric cavity volume 30–50 ML. And then all patients were treated with modified Roux-en-Y gastric bypass procedures. The jejunum was cut 75–100 cm from the Treitz ligament, and then the distal end of the jejunum was connected to the residual stomach or esophagus by an end-to-end or end-to-side anastomosis. The distal jejunum at 75–100 cm was connected to the proximal jejunum by an end-to-side anastomosis.

### 2.3. Observations

Body mass index (BMI), fasting blood glucose (FBG), two-hour postprandial blood glucose (2hPBG), hemoglobin A1c (HbA1c), fasting glucagon-like peptide-1 (GLP-1), two-hour glucagon-like peptide-1 (2hGLP-1), fasting gastric inhibitory polypeptide (FGIP), and two-hour gastric inhibitory polypeptide (2hGIP) levels were determined before surgery (0 months) and 1 week and 1, 3, and 6 months after surgery, respectively.

### 2.4. Statistical Analysis

The measurement data were expressed as the mean ± SD. Statistical analyses were performed by the *t* test using SPSS17.0 (SPSS Inc., Chicago, IL, USA). *P* < 0.05 was considered statistically significant.

## 3. Results

**Figure 1:**
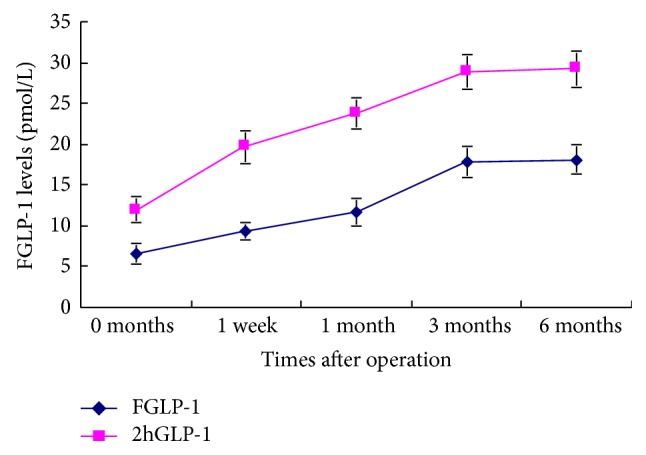
FGLP-1 levels before and after the surgery.

**Figure 2:**
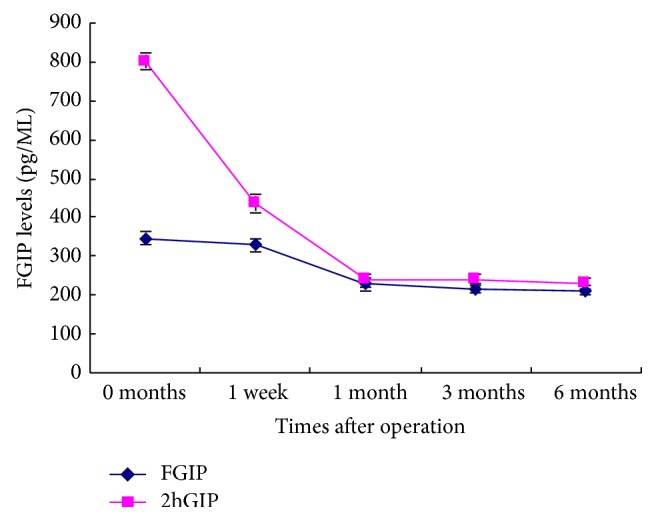
FGIP levels before and after the surgery.

Compared with the preoperative FBG and 2hPBG levels, these levels decreased 1, 3, and 6 months after surgery (*P* < 0.01). HbA1c levels were significantly decreased 3 and 6 months after surgery (*P* < 0.05). FGLP-1 and 2hGLP-1 levels were significantly increased at each time point after surgery (*P* < 0.05). Compared to FGLP-1 levels, the levels of 2hGLP-1 were significantly higher at the same time point (*P* < 0.01). FGIP levels were significantly decreased 1, 3, and 6 months after surgery (*P* < 0.05) and 2hGIP levels were significantly decreased at each time point after surgery (*P* < 0.01). Compared to FGIP levels at the same time point, the levels of 2hGIP were significantly higher before surgery (*P* < 0.01), but no significant differences were found between the two groups after surgery (Tables 1 and 2, Figures 1 and 2).

## 4. Discussion

Type 2 diabetes is a chronic metabolic syndrome marked by high levels of glucose in the blood. Owing to its various etiological factors and complicated mechanisms, the traditional way of treatment to control blood glucose is lack of stability, unable to suppress the disease development fundamentally; therefore, curing T2DM has become an urgent global medical goal [11].

Modified gastric bypass surgery began to be used in T2DM patients in the 1990s, and the efficiency of this procedure to cure obese patients with T2DM is between 86.6 and 91% [3–10]; previous research showed that 83%–86% of T2DM patients maintained normal levels of blood glucose following operation [12, 13]. In a follow-up study, American Diabetes Association [14] showed that this surgical procedure was also efficacious in T2DM patients without obesity.

In the present study, we retrospectively analyzed blood glucose indexes in patients with T2DM and gastric cancer or simple T2DM underwent gastrectomy using modified Roux-en-Y gastric bypass procedures. We found that, compared with the levels before surgery, FBG and 2hPBG levels were significantly decreased 1, 3, and 6 months after surgery; HbA1c levels were significantly decreased 3 and 6 months after surgery. The metabolism of blood glucose index improved obviously; therefore, the modified digestive tract reconstruction can better control blood glucose in patients with T2DM, and the curative effect is exact and reliable. Although the modified gastric bypass surgery had a better hypoglycemic effect and lasted longer [15], the mechanism is not very clear.

At present, there are many hypotheses related to this mechanism, among which, “the foregut hypothesis” and “hindgut hypothesis” have received significant attention [16]. The “foregut hypothesis” considers that food in the duodenum and proximal jejunum stimulate the production of GIP, thus improving insulin resistance [17–19]. The “hindgut hypothesis” considers that chyme in the terminal ileum and colon promotes the secretion of GLP-1 and stimulates the secretion of glucose-dependent insulin, which improves the insulin effect and the proliferation of islet B cells [20–26].

In order to further clarify the effect of modified gastric bypass surgery on incretin secretion in patients with T2DM, we analyzed FGLP-1, 2hGLP-1, FGIP, and 2hGIP levels in patients with T2DM. We found that FGLP-1 and 2hGLP-1 levels were significantly increased at each time point after surgery. The level of 2hGLP-1 was significantly higher than the level of FGLP-1 at the same time point. This is consistent with the changes in the secretion of GLP-1 in the “hindgut hypothesis.”

Meanwhile, FGIP levels were significantly decreased 1, 3, and 6 months after surgery; 2hGIP levels were significantly decreased at each time point after surgery. Compared to FGIP levels at the same time point, the levels of 2hGIP were significantly higher before surgery, but no significant differences were found between the two groups after surgery. These data prove what the “the foregut hypothesis” said that food into the foregut stimulates the production of GIP. Following the modified digestive tract reconstruction procedure, the food does not enter the foregut, directly into the hindgut, and led to a decline in FGIP and 2hGIP levels. Therefore, modified Roux-en-Y gastric bypass surgery can promote GLP-1 secretion in patients with T2DM, while reducing the secretion of GIP, thus resulting in better regulation of blood glucose [27].

## Figures and Tables

**Table 1:** Comparison of glucose metabolism index before and after the surgery.

	*n*	BMI	FBG	2hPBG	HbA1c
0 mo	50	25.23 ± 1.21	10.3 ± 1.1	14.1 ± 2.8	9.8 ± 1.1
1 wk	50	24.82 ± 1.16	9.2 ± 1.3	13.2 ± 2.5	9.7 ± 1.2
1 mo	50	24.35 ± 1.27	6.5 ± 1.5^#^	8.1 ± 1.9^#^	9.0 ± 1.1
3 mo	50	24.03 ± 1.15	5.5 ± 1.4^#^	6.2 ± 1.5^#^	6.7 ± 1.3^*∗*^
6 mo	50	23.87 ± 1.22	5.0 ± 1.1^#^	6.5 ± 1.8^#^	5.1 ± 1.2^#^

Data are shown as mean ± SD, BMI (kg/m^2^), FBG, and 2hPBG (mmol/L), HbA1c (%).

Compared with the before surgery (0 months) group ^*∗*^ *P* < 0.05, ^#^ *P* < 0.01.

**Table 2:** Comparison of GLP-1 and GIP levels before and after the surgery.

	*n*	FGLP-1	2hGLP-1	FGIP	2hGIP
0 mo	50	6.5 ± 1.3	11.9 ± 1.6^#^	347 ± 16	801 ± 21^#^
1 wk	50	9.3 ± 1.1^*∗*^	19.7 ± 2.0^▲#^	328 ± 15	436 ± 25^▲^
1 mo	50	11.6 ± 1.7^▲^	23.7 ± 1.9^▲#^	229 ± 16^*∗*^	238 ± 17^▲^
3 mo	50	17.9 ± 1.9^▲^	28.9 ± 2.1^▲#^	217 ± 12^*∗*^	241 ± 15^▲^
6 mo	50	18.1 ± 1.8^▲^	29.2 ± 2.2^▲#^	213 ± 14^*∗*^	230 ± 13^▲^

Data are shown as mean ± SD, FGLP-1 and 2hFGLP-1 (pmol/L), FGIP, and 2hFGIP (pg/ML).

Compared with the before surgery (0 months) group ^*∗*^ *P* < 0.05, ^▲^ *P* < 0.01.

Comparison of the same point in time ^#^ *P* < 0.01.
